# Pathway-like Activation of 3D Neuronal Constructs with an Optical Interface

**DOI:** 10.3390/bios15030179

**Published:** 2025-03-12

**Authors:** Saeed Omidi, Yevgeny Berdichevsky

**Affiliations:** 1 Department of Bioengineering, Lehigh University, Bethlehem, PA 18015, USA; sao221@lehigh.edu; 2 Department of Electrical and Computer Engineering, Lehigh University, Bethlehem, PA 18015, USA

**Keywords:** 3D, axons, brain-on-a-chip, microchannel, neuron, optical stimulation, optogenetics, pathway

## Abstract

Three-dimensional neuronal organoids, spheroids, and tissue mimics are increasingly used to model cognitive processes in vitro. These 3D constructs are also used to model the effects of neurological and psychiatric disorders and to perform computational tasks. The brain’s complex network of neurons is activated via feedforward sensory pathways. Therefore, an interface to 3D constructs that models sensory pathway-like inputs is desirable. In this work, an optical interface for 3D neuronal constructs was developed. Dendrites and axons extended by cortical neurons within the 3D constructs were guided into microchannel-confined bundles. These neurite bundles were then optogenetically stimulated, and evoked responses were evaluated by calcium imaging. Optical stimulation was designed to deliver distinct input patterns to the network in the 3D construct, mimicking sensory pathway inputs to cortical areas in the intact brain. Responses of the network to the stimulation possessed features of neuronal population code, including separability by input pattern and mixed selectivity of individual neurons. This work represents the first demonstration of a pathway-like activation of networks in 3D constructs. Another innovation of this work is the development of an all-optical interface to 3D neuronal constructs, which does not require the use of expensive microelectrode arrays. This interface may enable the use of 3D neuronal constructs for investigations into cortical information processing. It may also enable studies into the effects of neurodegenerative or psychiatric disorders on cortical computation.

## 1. Introduction

Three-dimensional (3D) neuronal constructs, which include spheroids, organoids, assembloids, and tissue mimics, are increasingly used to model neurological and psychiatric disorders [1,2,3]. These in vitro models are used to understand the basic pathological mechanisms and to accelerate drug development for disorders such as Alzheimer’s disease, Parkinson’s disease, epilepsy, and schizophrenia, among others [4,5,6]. There is also an increased interest in using 3D neuronal constructs to study cognition [7] and to use them as biological computers [8], with the goal of improving the energy efficiency of computing [9]. Three-dimensional systems are gaining in popularity since they contain neurons in a more realistic, brain-like environment compared to 2D models. This helps neural networks embedded in 3D constructs to achieve more brain-like activity and drug response [10,11]. However, the use of 3D neuronal systems brings up unique challenges, which are described below.

Clinical symptoms of neurological and psychiatric diseases reflect the disturbance in information processing by the brain’s networks of neurons due to disease-induced changes [12,13,14]. Information processing can be defined as the relationship between network input and output. Networks contained in 3D neuronal constructs can also be defined by their input and output, with input represented by stimulation of neurons and output represented by evoked neural activity. Neuronal constructs are used to model disease by experimentally altering gene expression, applying toxins, or delivering physical damage. The effect of these experimental manipulations on the network input–output relationship may model effects of disease on the networks in the patient’s brain.

Detection of deficits in the input-output relationship in 3D constructs is thus essential to their application as neurological and psychiatric disease models. This motivated the development of sophisticated methods of stimulating and detecting neural activity in 3D tissues. Recently developed technologies include 3D multiple electrode arrays (MEAs) and conformal, flexible, and cup electrodes for spheroids and organoids [15,16,17]. These methods achieve the goal of stimulating neurons within the 3D constructs by delivering spatially confined currents to the surface or the interior of the construct. However, due to the structure of 3D constructs, which contain neural soma entangled with dendrites and axons (together termed ‘neurites’), stimulation activates these sub-cellular portions of neurons indiscriminately. Neuronal activation in the intact brain occurs via a starkly different mechanism. Information, such as a sensory stimulus, travels via axonal pathways from sensory organs to sub-cortical structures and then to cortical areas [18,19]. Information is transmitted by action potential firing rates in individual axons that make up the pathways. Postsynaptic neurons then change their firing rates based on the integration of inputs that arrive via different synapses [20]. The collection of firing rates of neurons in a cortical area at a given time is a population code that represents specific features of the sensory stimulus [7,21,22].

The organization of the brain’s circuitry into pathways has been modeled in vitro by placing neurons into polydimethylsiloxane (PDMS) wells connected by microchannels [23,24]. Neurons sprout axons and dendrites (collectively termed neurites), which then grow along microchannels and form functional synapses with neurons in the connected well. Microchannel-confined neurites are capable of transmitting spontaneous and evoked neuronal activity [25,26,27]. Microelectrodes placed under the microchannels [28] have been used to stimulate neurite bundles directly. This technique was used to study the propagation of action potentials in axons [25], propagation of patterns, plasticity, and non-linearity of response in modular networks [29,30], and intracellular dynamics in postsynaptic neurons [31]. Stimulation of axons linking groups of hippocampal neurons was used to analyze pattern separation and completion, with ‘one-hot’ patterns encoded as stimulations of different neurite bundles [32]. The population code of postsynaptic neurons represented a transformation of the input encoding, enabling the study of information processing in hippocampal pathways. These studies have been carried out using 2D networks of neurons.

3D neuronal constructs and tissues can also be placed in PDMS wells connected by microchannels [23,27]. Neurites sprouted by neurons exit the confines of 3D constructs, enter microchannels, and form synaptically connected networks. This type of system could be termed 2.5D, as it is composed of 3D aggregates containing neural soma and neurites extended beyond the boundaries of the aggregate on a 2D surface. These systems have a high potential for modeling healthy and defective information processing: neurons are placed in a brain–like 3D environment, while axons and dendrites are easily accessible in 2D and may be used to deliver encoded stimuli in a manner that mimics the functionality of the brain’s pathways. To the best of our knowledge, this potential has not yet been realized in an experimental system.

In this work, we demonstrate, for the first time, that neurites extended from 3D cortical aggregates can be used for interfacing with the neurons within the 3D aggregate. We used 3D constructs made of scaffold-free, aggregated cortical neurons. These constructs possess a cortex-like density of neurons and a strong network of synaptic connections [33]. We elected to stimulate neurites via optogenetics rather than electrical pulses due to the cell specificity of this optical method. We also used optical detection of neuronal activity via genetically expressed calcium indicator to create an all-optical interface. This work represents the first time that an all-optical interface was used to access networks in 3D neuronal constructs. This interface does not require the use of a cleanroom to fabricate customized MEA chips, which may be unavailable to neuroscience laboratories. We demonstrate that the delivery of stimulation patterns to neurites, which mimics the way that neurons in each stage of the neuronal pathway are activated in the brain, evokes separable and classifiable population codes in the neuronal network contained in the 3D construct.

## 2. Materials and Methods

### 2.1. Fabrication of the PDMS Devices

**Figure 1:**
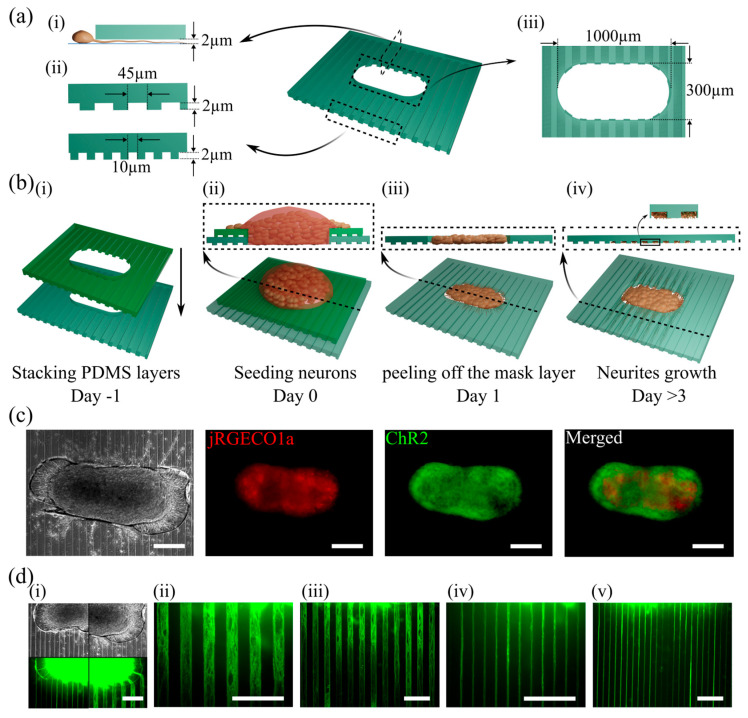
PDMS device *for culture of 3D cortical constructs with guided neurites in microchannels*. (**a**) Schematics showing geometrical features of the PDMS device. (**i**) A longitudinal cross section containing one microchannel. The height of microchannel prevented neuron soma from entering but permitted neurite extension. (**ii**) Cross sections of two different devices used in this study. (**iii**) The size of the well for cell seeding. (**b**) Schematic showing the cell seeding protocol: (**i**) two PDMS layers (bottom one is the main layer and the top one serves as the mask layer) are stacked one day before cell seeding; (**ii**) high-density solution of dissociated neurons is placed into the well of the double-layer PDMS device; (**iii**) mask layer is peeled off after 24 h, removing excess cells; (**iv**) neural processes growing through channels during maturation. (**c**) Phase contrast and fluorescent images showing jRGECO1a expression in neural cells and Channelrhodopsin2 (ChR2) expression in axons and dendrites. (**d**) Axons enter and fill the channels: (**i**) phase contrast images (**top**) and fluorescent images of ChR2 expression (**bottom**) in devices with narrow (**left**) and wide (**right**) microchannels; (**ii**,**iii**) show 20X and 10X magnification images of wide channels with axons; (**iv**,**v**) show 20X and 10X magnification images of narrow channels with axons. All scale bars are 200 µm.

SU-8/silicon mold masters featuring negative-relief arrays of microchannels were utilized. Mold masters were fabricated on silicon wafers using SU-8 negative photoresist. A 2 µm thick layer of SU-8 2 (Kayaku Advanced Materials, Westborough, MA, USA) was spun onto a 3-inch silicon wafer. The wafer was then exposed to ultraviolet (UV) light through a mask containing the negative of the desired channel array. Resist was developed using SU-8 developer (Kayaku Advanced Materials, Westborough, MA, USA). Two separate molds were utilized: one with microchannels of 45 µm width and the other with microchannels of 10 µm width. A 10:1 ratio of Polydimethylsiloxane (PDMS) base and curing agent (Sylgard 184, Electron Microscopy Sciences, Hatfield, PA, USA) was mixed at 10:1 ratio and coated on the SU-8 master at 500 rpm using a spin coater (Laurell, Landsdale, PA, USA) for 3.5 min, then baked on a hotplate at 120 °C for 5 min and cured at 75 °C overnight. The PDMS layer was then carefully peeled off from the master. A rectangular well (about 300 µm × 1000 µm) was punched in the center of the PDMS to create the 3D culture space. Two PDMS layers, the top one serving as a mask layer with slightly smaller dimensions, were attached to the surface of a poly-D-lysine (PDL, Sigma-Aldrich, St. Louis, MO, USA) coated Petri dish. Figure 1a shows the well and microchannel dimensions. Microchannels had a depth of 2 µm, which prevents the entry of neural somas into the channels, allowing only neurites to pass through.

### 2.2. Cell Culture

The PDMS device was submerged under neural culture medium (97.5% Neurobasal-A, 2% B27, 1 mM GlutaMAX, and 30 µg/mL Gentamycin (all from ThermoFisher Scientific)) and incubated overnight at 37 °C and 5% CO_2_. Primary rat cortical neurons were prepared from a post-natal day 0–1 Sprague Dawley rat pup (Charles River) by dissecting and dissociating the cortex, following a previously published protocol [34]. All animal use protocols were approved by the Institutional Animal Care and Use Committee (IACUC) at Lehigh University and conducted under the United States Public Health Service Policy on Humane Care and Use of Laboratory Animals. After centrifugation, the dissociated neurons were resuspended in approximately 10 µL of neural culture medium to create a highly dense cell suspension. About 2 µL of this cell suspension, containing about 5–6 × 10^5^ neurons, was seeded into the well of the PDMS device. The device was then incubated for 15 min to let the cells settle before submerging under the culture medium. The following day, the top PDMS mask layer was carefully peeled off to remove any neurons on the top surface of the device and not in the well. Devices with cells were maintained in the incubator, with the culture medium changed 3 times per week. Figure 1(b(ii–iv)) shows the cell seeding procedure.

### 2.3. Optical Recording and Optogenetic Stimulation

**Figure 2:**
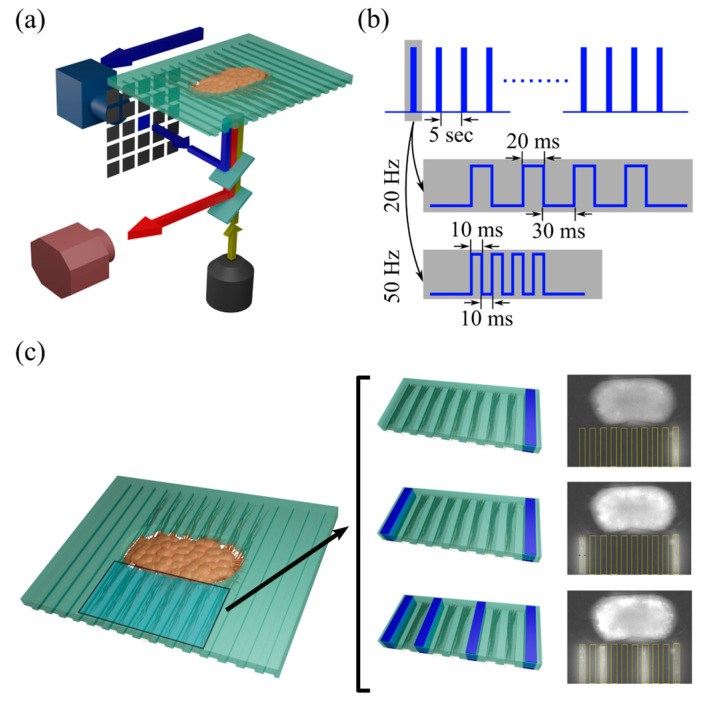
Optical stimulation and detection of neural activity. (**a**) Experimental setup, including the device with culture and a dual light path to guide fluorescent light (red) to the camera and stimulation light (blue) from patterned illuminator to the device. Excitation light (yellow) is provided by a dedicated light source. (**b**) Waveforms for 20 Hz and 50 Hz optical stimulation with blue light pulses. (**c**) Schematic of the device shows 1, 2, or 4 channels as stimulation ROIs (inputs), along with actual video frames for each stimulation condition.

**Figure 4:**
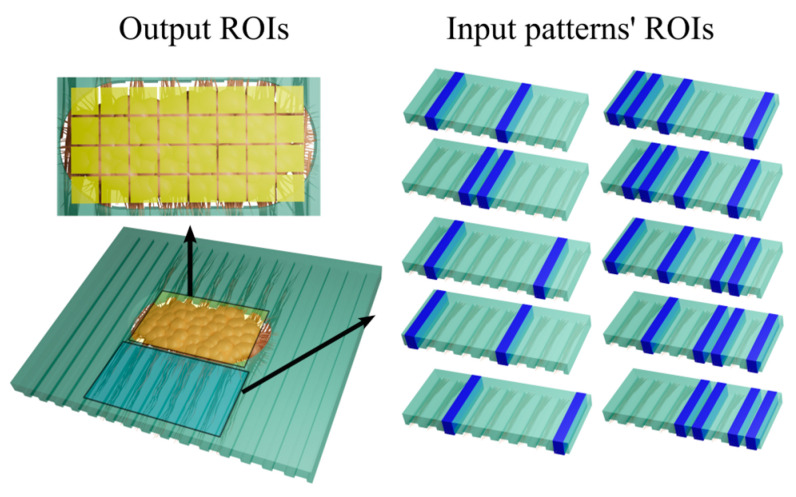
A schematic of the device showing the 5 stimulation patterns with 2 or 4 channels. Output ROIs are also shown.

**Figure 5:**
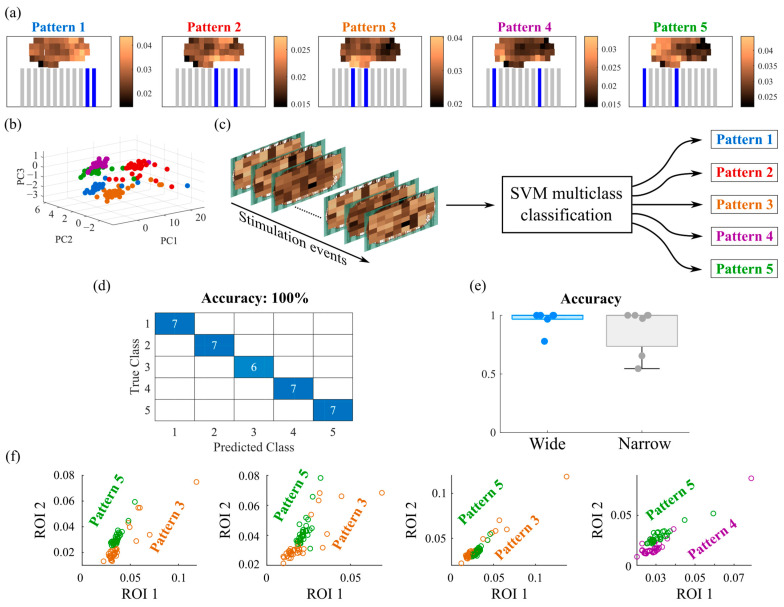
Analysis of responses evoked by multi-pattern stimulation. (**a**) Average evoked ΔF/F in each ROI by 5 stimulation patterns in a representative experiment. (**b**) Individual evoked responses in a representative experiment plotted on axes corresponding to first 3 principal components (PCs). Colors correspond to stimulation patterns in (**a**). (**c**) The pipeline of SVM multiclass classification using output ROIs for each stimulation event as inputs (SVM predictors). (**d**) Representative confusion matrix for accuracy of test data classification for experiment in (**a**). (**e**) Classification accuracies for all experiments with wide- and narrow-channel devices are plotted as dots, and box plots show the median and quartiles (*n* = 5 experiments for wide-channel and *n* = 6 experiments for narrow-channel devices). (**f**) Example responses of ROIs to different patterns demonstrate mixed selectivity. Changes in fluorescence in one ROI are not sufficient to infer a stimulus pattern, but when changes for 2 ROIs are plotted together, clustering of responses by pattern becomes apparent.

On day-in vitro (DIV) 1, the culture medium was replaced with medium containing adeno-associated virus (AAV) particles, expressing a genetically encoded intercellular calcium indicator jRGECO1a (a gift from Douglas and Kim GENIE, Addgene plasmid # 100854, Watertown, MA, USA) under the Synapsin promoter for optical recording. For optogenetic stimulation, cells were infected with AAV to express light-sensitive transmembrane Channelrhodopsin2 (ChR2, a gift from Karl Deisseroth, Addgene plasmid # 26973, Watertown, MA, USA). To activate specific regions of interest (ROIs), blue light pulses (480 nm wavelength, 10 mW/mm^2^ power) were generated using an LED light source (Mightex, part #: GCS-0470-50-A510, Pleasanton, CA, USA) and delivered through a digital micromirror device-based pattern illuminator Polygon400 (Mightex, Pleasanton, CA, USA). Figure 1c shows the brightfield image of culture (DIV 25), jRGECO1a expression in neural somas, and ChR2 expression in neural processes and axons. Figure 1d shows the axon bundles entering the wide and narrow microchannels, starting from DIV 3. Microchannels that were filled with neurites expressing ChR2 served as stimulation ROIs. The number of microchannels available for stimulation was 11–12 for wide channel devices and 19 to 21 for narrow channel devices. Stimulation ROIs were drawn around the selected microchannels with the same width as a single channel and the length of ~300 µm. ROIs were offset from the soma compartment by ~40 µm. ROI area was 1.35 × 10^4^ µm^2^ and 0.3 × 10^4^ µm^2^ for wide and narrow channel devices, respectively. Examples of stimulation patterns composed of one or more ROIs are shown in Figures 2c, 4 and 5a. The PDMS device containing neural culture was placed in a mini-incubator mounted on a dual-deck fluorescent microscope (IX73, Olympus, Center Valley, PA, USA). Environmental conditions were maintained at 37 °C using a temperature controller (Warner Instruments, Holliston, MA, USA) and neutral pH with humidified mixed blood gas (Airgas, part #: Z03NI7442000074, composed of 5% CO_2_, 21% O_2_, and balanced N_2_). Neural activity, represented by changes in jRGECO1a fluorescence, was recorded using a Quantalux Monochrome sCMOS camera (Thorlabs, Newton, NJ, USA) at 20 frames per second (fps). Figure 2a shows the experimental setup. Light from the LED fluorescence illuminator (Excelitas X-cite 110LED, Pittsburgh, PA, USA) passed through the excitation filter (580 nm center wavelength) and was delivered to the culture simultaneously with blue stimulation light pulses from the pattern illuminator, via the lower and upper decks of the microscope, respectively. Emitted jRGECO1a fluorescence was filtered (610 nm center wavelength) and delivered to the camera. The stimulation consisted of a train of 4 pulses repeated every 5 s for a total of 40 train repeats during a single experiment. The experiments were conducted using devices with both wide and narrow channels. Two stimulation protocols were used, with train frequencies of 20 Hz and 50 Hz, targeting 1, 2, or 4 channel(s) as stimulation ROIs. Figure 2b provides an overview of the stimulation protocol. Figure 2c depicts a schematic of the device, alongside schematics and actual video frames of 1, 2, and 4 channel stimulations.

### 2.4. Neural Activity Analysis

The recorded videos were analyzed by FIJI ImageJ software (version 2.14.0/1.54f). The well seeded with cells (neural culture region) was divided into an array of regions of interest (ROIs), and the raw mean gray values of all ROIs were extracted. Using an asymmetric least square smoothing method [35], slow variation in the fluorescence background was removed by assuming an overlayed smooth baseline combined with asymmetric weighting deviations from the smooth trend. Change in fluorescence relative to baseline, normalized by baseline, was then calculated for each ROI (ΔF/F).

## 3. Results

### 3.1. Stimulation of Axon Bundles in Microchannels Evoked Responses in the 3D Neural Culture

**Figure 3:**
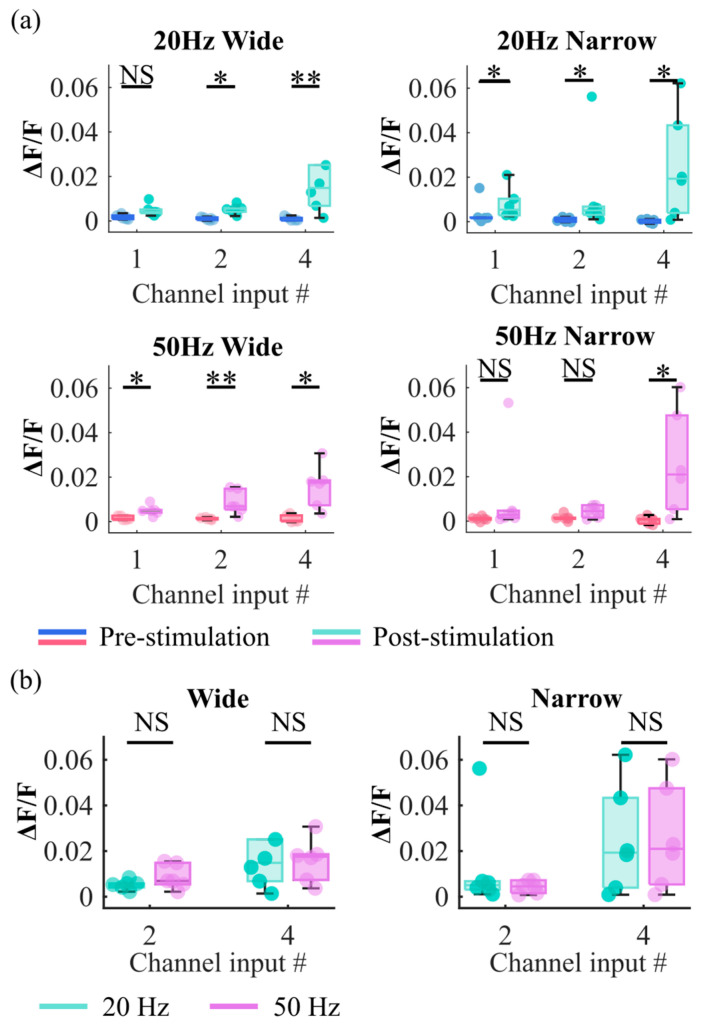
Analysis of the evoked responses. (**a**) Comparison of ΔF/F pre- and post-stimulation. Each circle represents the median of all stimulation events for an experiment (*n* = 6 experiments per stimulation condition). (**b**) Comparison of 20 Hz and 50 Hz stimulation for 2 and 4 channels. Two sample KS-test significance values shown as: * *p* < 0.05, ** *p* < 0.01, NS means Not Significant. # stands for ‘number’.

Stimulation with 20 Hz and 50 Hz pulse trains was delivered to devices with wide and narrow microchannels. Stimulation patterns targeted one, two, or four channels (Figure 2c). To determine whether the stimulation via microchannels successfully evoked a response in the neural culture, pre-stimulation and post-stimulation ΔF/Fs were compared. Stimulation events that either caused a burst response or were applied during the culture’s bursts of spontaneous activity were excluded from the analysis. One frame (50 ms) before the stimulation was used to determine pre-stimulation ΔF/F, and the second frame after the end of stimulation (100 ms later) was used to determine post-stimulation ΔF/F. Pre- and post-stimulation neuronal activity in devices with different channel widths and stimulated with trains of different frequencies are shown in Figure 3a. A Kolmogorov–Smirnov (KS) test was employed to statistically compare pre- and post-stimulation ΔF/Fs for each condition. This analysis demonstrated that stimulation of axon bundles through microchannels successfully evoked responses in the 3D neural cultures. Stimulation delivered to four microchannels simultaneously was successful in evoking a response in 3D culture for all device and train frequency conditions, while stimulation delivered to 2 microchannels was successful in all but one condition. Post-stimulation ΔF/F data for 20 Hz and 50 Hz stimulation frequencies were compared, focusing on conditions with 2 and 4 stimulation channels. Figure 3b presents this comparison, indicating that no significant differences in evoked responses between 20 Hz and 50 Hz stimulations were found (Figure 3b). Since the 50 Hz stimulation protocol enabled the delivery of a 4-pulse train in a shorter period of time compared to the 20 Hz protocol, the 50 Hz protocol was selected for all subsequent experiments.

### 3.2. Responses Evoked by Different Stimulation Patterns Are Separable and Classifiable

A multi-pattern stimulation approach was used to investigate evoked responses from different regions of neural culture. This was achieved by selecting five patterns of stimulation using two or four microchannels as inputs, with each pattern delivered 40 times. Figure 4 presents a schematic of the device output ROIs in the 3D culture. Initially, pre- and post-stimulation ΔF/Fs were analyzed to determine if all stimulation patterns successfully evoked responses, similar to the analysis shown in Figure 3a. This initial analysis did not differentiate between the patterns of stimulation. Figure S1 shows the result of this analysis and confirms that these stimulations did evoke successful responses. To determine if the evoked responses were classifiable based on the stimulation patterns, the average post-stimulation ΔF/Fs for all events were calculated for each output ROI and mapped to their physical locations (Figure 5a, Supplementary Videos S1–S4). These maps indicated that the evoked responses varied by location of ROI and depended on the stimulation pattern used. Principal Component Analysis (PCA) was applied to reduce the dimensions of the output ROIs to three principal components (Figure 5b). Individual evoked responses clustered by stimulation pattern, demonstrating separability in this low-dimensional space. A decoder based on a multiclass Support Vector Machine (SVM) with a linear kernel and a one-vs-all error-correcting output code approach was employed to determine whether output activity could be used to infer the input pattern (Figure 5c). A 5-fold cross-validation strategy with 20% of the data reserved for testing were used. Figure 5d displays the confusion matrix and accuracy for the test data. The SVM used five binary learners, each designed to differentiate one class (pattern) from the others, identifying optimal support vectors for classification. Each learner produced a vector with linear predictor coefficients, β, proportional to the number of predictors (output ROIs). Figure S2a compares the average post-stimulation ΔF/Fs, observed in Figure 5a, with the β coefficients for the same class plotted against the output ROIs, confirming that the β coefficients reflect the actual post-stimulation ΔF/Fs. Furthermore, Figure S2b maps these β coefficients to their corresponding output ROIs. High classification accuracy was achieved for devices with both wide and narrow channels (Figure 5e). Supplementary videos S1 and S2 (for wide channel device) and S3 and S4 (for narrow channel device) show representative multi-pattern experiments. Heatmaps, representing ΔF/Fs of output ROIs, were superimposed on the videos S1 and S3 and shown as videos S2 and S4. Videos S1 and S3 show raw data. We then examined whether ROIs selectively responded to a single input pattern or displayed ‘mixed selectivity’ by responding to multiple input patterns. The latter case is similar to evoked responses in the intact cortex, where neurons respond to multiple stimuli, and population decoding themes are required to infer stimulus from neural activity [36]. We found that ROIs demonstrated mixed selectivity by responding to multiple patterns, and analysis of multiple ROIs was required to infer stimulation pattern (Figure 5f).

## 4. Discussion

In this study, we introduced a novel device designed to model cortical pathway-like inputs to a network of cortical neurons. The device includes an optical interface to deliver patterned stimuli to the neural network through axons and dendrites. This device functions as a miniaturized cortical network on a dish, making it ideal for the investigation of cortical information processing. The dense cortical construct within the device maintains its 3D structure over time, with neurons within the 3D environment establishing robust synaptic connections. The device’s microchannels organize axons and dendrites extended by the neuron within the 3D construct into independent bundles separate from the construct (Figure 1(a(i),d)). The cortical culture and neural processes in microchannels remained healthy (based on their spontaneous activity and responsiveness to stimulation) for at least 6 weeks, demonstrating that the 3D construct maintains its integrity and circuit connectivity over a longer period of time than 2D neural cultures.

Optogenetic stimulation of axons expressing ChR2 is widely used to map pathways in the intact brain [37]. A group of neurons sharing a genetic identity or a common physical location are made to express ChR2, and then light is delivered to the area of the brain containing their axons to determine the presence of functional synapses. In this type of mapping, stimulation light is delivered to a large area to stimulate many axons simultaneously, causing detectable changes in the activity of postsynaptic neurons. Localized stimulation, using a focused laser beam, for example, is also used for mapping; however, in this case, the detection of synaptic currents via whole-cell recordings or other methods is required to confirm the presence of a connection. In this work, we delivered light to a narrow area containing just a few neurites in one, two, or four microchannels. We have confirmed that such localized stimulation causes significant changes in neuronal activity assessed with the fluorescence of a calcium indicator. The strong effect of localized stimulation may be due to the presence of ChR2-expressing dendrites as well as axons in the stimulated area. Strong connectivity between neurons in 3D constructs may also amplify input stimulation. Together, these findings confirmed the feasibility of using neurites extended by neurons in the 3D construct for stimulation.

We used the fluorescence of a genetically encoded calcium indicator jRGECO1a to assess neuronal activity in the 3D construct. Fluorescence changes in jRGECO1a reflect the increase or decrease in intracellular calcium concentration ([Ca^2+^]_i_) in neurons. In a resting (inactive) neuron, calcium concentration is low. Calcium enters neurons during an action potential and is then slowly pumped out of the neuron, decaying to a baseline, low concentration. Subsequent action potentials may result in an accumulation of [Ca^2+^]_i_ if occurring at shorter intervals than the time constant of the calcium decay [38]. The canonical view of neuronal calcium dynamics is that the calcium level in a neuron represents the convolution of the calcium kernel (reflecting its rise and decay time constants) with a train of action potentials. In this view, the timing of action potentials can be extracted from [Ca^2+^]_i_ signal by deconvolving it with [Ca^2+^]_i_ kernel [39]. This process is accurate for neurons that are mostly at rest and fire action potentials occasionally. However, accuracy in determining action potential timing drops significantly for neurons that are active throughout the experiment and thus possess an average [Ca^2+^]_i_ that is significantly different than the baseline. In this case, stimuli may result in a decrease as well as an increase in [Ca^2+^]_i_ relative to the average. These changes may not be accurately modeled by convolution with [Ca^2+^]_i_ kernel since the average firing rate for a neuron in an active network may not be known. In an active network, assessment of neuronal activity can be carried out directly via [Ca^2+^]_i_ levels without deconvolution [40]. Removal of the requirement for deconvolution also enables analysis of activity in the neuropil (composed of a dense network of axons and dendrites), which can be as sensitive to stimulation features as activity in the neuronal soma [41] and encode information about cognitive processes [42]. In our study, we defined output ROIs in the 3D construct such that they included both neuronal soma and neuropil and assessed activity without deconvolution.

Cortical areas use population codes to encode features of visual, somatosensory, auditory, and other stimuli [21,22]. Encoding is analyzed by constructing population activity vectors, representing firing rates or [Ca^2+^]_I,_ in many neurons imaged in parallel at a given point in time. Dimensions of population activity vectors are reduced using techniques such as PCA and plotted in low-dimensional space [43]. The separability of population activities corresponding to different stimuli (such as different images, surface textures, or sounds) is interpreted as the ability of the underlying neuronal population to categorize these stimuli via population code [22,44]. Analysis of population codes in different areas of the cortex revealed that many neurons have so-called ‘mixed selectivity’. These neurons significantly modulate their activity in response to more than one stimulus category, and population-level code is required to accurately classify stimuli [21,36]. We interpreted these aspects of the intact cortical information processing in the context of our in vitro system as follows. Different stimuli were encoded as different groupings of neurites receiving optogenetic activation at a given time, mimicking activation patterns in thalamo-cortical and cortical-cortical pathways [44,45]. Population activity vectors were constructed from Ca^2+^ levels in different ROIs at time points immediately following the delivery of stimuli. We found that these population activity vectors were separable in low dimensional space, that stimulus patterns could be classified via population activity vectors, and that individual ROIs were characterized by ‘mixed selectivity’. These results demonstrate that we could model these aspects of cortical information processing in vitro.

Previous in vitro patterned stimulation experiments, whether with stimulation via electrodes or optical patterns, were carried out in 2D networks of neurons, with stimulation delivered directly to cell bodies [46,47,48]. In this work, we aimed to place information processing elements of the network, including neurons and neuropil, into 3D constructs, where densities of connections and interactions between neurons, astrocytes, and extracellular matrix could occur in a manner that is closer to the environment found in the intact cortex. Our results from the stimulation of microchannel-confined neurites demonstrate the feasibility of delivering patterned stimuli to 3D constructs. For wide-channel devices, there were 12 available channels for neural processes to grow into, leading to 66 possible combinations of selecting 2 channels out of 12, and 495 combinations for selecting 4 channels based on the combination theory. For the narrower channels, there were between 19 and 23 channels, with some devices having few blocked channels or very few neural processes growing into them, which could be omitted from stimulation patterns. We can similarly calculate that possible combinations of selecting 2 or 4 channels among the average 21 channels available are 210 and 5985, respectively. These large numbers of possible stimuli will enable future studies of fundamental features of cortical information processing, such as the dimensionality of neural representations [36].

Neuronal pathways in the brain are composed of hundreds or thousands of independent axons. This is an order of magnitude higher number of connections than the number of channels in the device reported in this work. The number of independent neuronal connections in the device may be improved in future work by using microchannels of smaller width. However, there may be a fundamental limitation to the minimum width of a microchannel: smaller channels will contain fewer axons, and the efficiency of optical stimulation delivered to microchannels may be lower. The lower limit on the channel width may need to be determined in future studies. Another limitation of current work is that the microchannel stimulation activates both dendrites and axons extended by neurons within a single 3D construct. In future work, this limitation may be overcome by utilizing multiple 3D constructs connected by longer microchannels such that dendrites and axons can be separated.

## 5. Conclusions

We developed a novel in vitro model of pathway inputs to the cortex. The model features a 2D optical interface to a cultured 3D cortical network. Features of the model include: (1) a dense synaptically connected network of cortical neurons in a 3D environment, (2) guidance of sprouted axons and dendrites into microchannel-confined bundles, and (3) an optical interface capable of stimulating a 3D network with well-defined input patterns and detecting the activity of neuronal populations. This model may serve as a tool for fundamental investigations into population-level cortical information processing. It may also enable studies into the effects of neurodegenerative or psychiatric disorders on cortical computation.

## Data Availability

The data supporting the conclusions of this article will be made available by the authors on request.

## Supplementary Materials

The following supporting information can be downloaded at: https://www.mdpi.com/article/10.3390/bios15030179/s1, Figure S1: Analysis of post-stimulation ΔF/F for multi-pattern stimulation shows the successful evoked response; Figure S2: Comparison between the SVM β coefficient for each classifier and the average of post-stimulation ΔF/F plotted versus ROIs; Video S1: This video shows an example of evoked activity in a wide-channel device for the multi-pattern experiment. Video S2: This video shows a heatmap, representing ΔF/Fs of output ROIs, superimposed on the raw data (which is Video S1); Video S3: This video shows an example of evoked activity in a narrow-channel device for the multi-pattern experiment; Video S4: This video shows a heatmap, representing ΔF/Fs of output ROIs, superimposed on the raw data (which is Video S3).
