# Supplementary material for: Pathway-like Activation of 3D Neuronal Constructs with an Optical Interface
Source: Biosensors (Basel). 2025 Mar 12;15(3):179. doi: 10.3390/bios15030179 (PMC11940104; doi:10.3390/bios15030179)
Supplement: Supplementary file 1 [file biosensors-15-00179-s001.zip › biosensors-3485295 - Supplementary File.pdf]

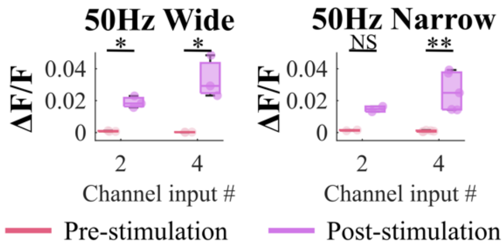

Figure. S1 Analysis of post-stimulation **Δ**F/F for multi-pattern stimulation shows the successful evoked response. KS-test was used here as the statistical test , * $p < 0.05$, ** $p < 0.01$, NS means Not Significant.

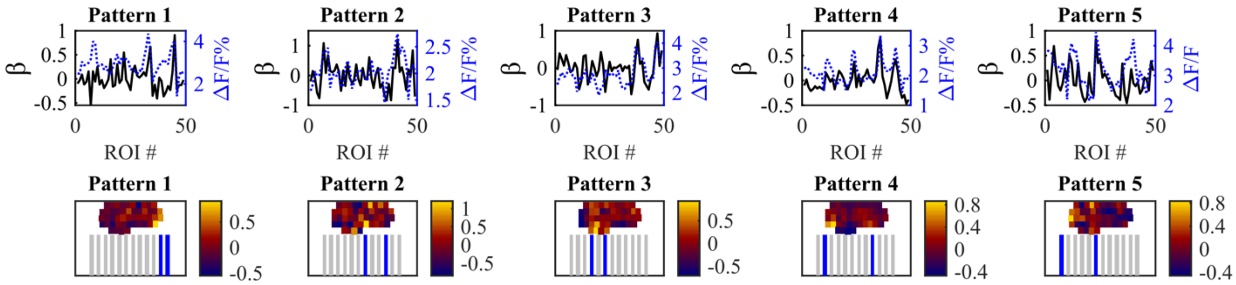

Figure. S2 Comparison between the SVM β coefficient for each classifier and the average of post-stimulation **Δ**F/F plotted versus ROIs. β coefficients are also plotted as a map OF ROIs for each pattern.
